# Discovery and Profiling of Protein Cysteine *S*-2-Carboxypropylation

**DOI:** 10.3390/molecules30214255

**Published:** 2025-10-31

**Authors:** Jiabao Song, Kejun Yin, Ronghu Wu, Y. George Zheng

**Affiliations:** 1 Department of Pharmaceutical and Biomedical Sciences, College of Pharmacy, University of Georgia, Athens, GA 30602, USA; jiabao0914@gmail.com; 2 School of Chemistry and Biochemistry, Georgia Institute of Technology, Atlanta, GA 30332, USA; kejun.yin@gatech.edu (K.Y.); ronghu.wu@chemistry.gatech.edu (R.W.)

**Keywords:** cysteine *S*-2-carboxypropylation (C2cp), PTM, methacrylyl-CoA, valine metabolism, chemical proteomics

## Abstract

Methacrylyl-CoA is a key metabolic intermediate in the valine catabolic pathway. Its accumulation has been found to be cytotoxic and associated with pathological conditions. Nevertheless, detailed biological effects of methacrylyl-CoA and methacrylate in human physiology and pathology are poorly understood. We propose that the electrophilicity of the alkene bond in the methacrylyl group can react with the cysteine residues in proteins resulting in an unexplored protein post-translational modification (PTM), cysteine S-2-carboxypropylation (C2cp). To test and validate this mechanistic hypothesis, we experimentally detected and profiled S-2-carboxypropylated proteins from the complex cellular proteome with the design and application of a bioorthogonal chemical probe, N-propargyl methacrylamide. We tested the probe in different mammalian cell models and demonstrated its versatility and sensitivity to protein cysteine S-2-carboxypropylation. We established quantitative chemical proteomics for global and site-specific profiling of protein S-2-carboxypropylation, which successfully identified 403 S-2-carboxypropylated proteins and 120 cysteine modification sites from HEK293T cells. Through bioinformatic analysis, we found that C2cp-modified proteins were involved in a variety of critical cellular functions including translation, RNA splicing, and protein folding. Our chemoproteomic studies demonstrating the proteome-wide distribution of cysteine S-2-carboxypropylation provide a new biochemical mechanism for the functional investigation of methacrylyl-CoA and understanding valine-related metabolic disorders.

## 1. Introduction

Branched-chain amino acids (BCAAs) that include leucine, isoleucine, and valine are essential building blocks for protein synthesis in all living organisms. BCAAs account for protein anabolic functionalities, energy production, signaling transmission, etc. [1,2,3]. Administration of leucine to food-deprived rats has been shown to effectively stimulate protein synthesis in skeletal muscles [4]. Administering leucine to rat muscle cells suppresses the activity of AMP-activated protein kinase (AMPK), which is known to maintain energy homeostasis in various tissues [5]. Isoleucine or valine deficiency reduces the fat mass in mice through promoting energy expenditure and modulating fat metabolism [6]. Recent studies on the isoleucine metabolism in mammalian cells suggest that it induces beta-defensins and demonstrates an immunotherapy effect on infectious disease [7]. Importantly, dysregulation of BCAAs has been found to associate with multiple diseases such as liver cirrhosis, diabetes, immune system disorders, and urine diseases [8,9]. For instance, BCAAs regulate the mTOR signaling pathway, a key therapeutic target in cancer biology [10]. Emerging roles of BCAAs in insulin resistance and strong correlation with obesity have also been revealed [11,12]. Biochemically, the catabolic pathways of BCAAs consist of multiple enzyme-mediated metabolic steps including reversible transamination, irreversible oxidative decarboxylation, and dehydrogenation [13,14]. The major end products (acetyl-CoA, succinyl-CoA) of BCAA metabolism further enter the Krebs cycle for energy production or participate in protein post-translational modifications [15,16,17].

Methacrylyl-CoA is a key intermediate metabolite generated in the valine degradation pathway. High levels of methacrylyl-CoA are toxic to cells and have been implicated in multiple valine metabolism-associated disorders [18,19]. Studies on the toxicity of methacrylyl-CoA can be traced back to the 1980s when it was found that an inherited mutation of 3-hydroxyisobutyryl-CoA hydrolase (HIBCH) enzyme led to a physical malformation and inborn error [20,21]. HIBCH deficiency or loss of the short-chain enoyl-CoA hydratase (ECHS1) activity generates excess methacrylyl-CoA, which is a causing factor for metabolic diseases such as liver dysfunction, impaired ATP production, and lactic acidosis [19,20,22]. It was further found that activities of methacrylyl-CoA hydratase and HIBCH decrease in livers with cirrhosis or hepatocellular carcinoma [23], indicating a decreased capability for detoxifying methacrylyl-CoA. The defect of ECHS1 in Leigh disease has been detected, which leads to the accumulation of methacrylyl-CoA and brain pathology [22]. From the chemical perspective, the double bond on methacrylyl-CoA owns a strong electrophilic nature and can undergo the thia-Michael addition to conjugate with a free thiol group [18]. Clinical studies show that N-acetyl-*S*-(2-carboxypropyl)cysteine, which is likely produced from methacrylyl-CoA, is a useful biomarker for the diagnosis of the ECHS1 deficiency (ECHS1D) [24,25]. A previous study has identified that, by incubating fibroblasts with either radioisotope-labeled valine or cysteine, patients with HIBCH mutations demonstrated an elevated secretion of *S*-2-carboxypropyl-cysteamine and *S*-2-carboxypropyl-cysteine molecules [21]. However, direct evidence for *S*-2-carboxypropylated proteins are unexplored. Recently, we and our colleagues found that as a key epigenetic enzyme, histone acetyltransferase 1 (HAT1) serves as a mitochondrial modulator [26] and catalyzes protein methacrylylation by transferring the methacrylic group from methacrylyl-CoA to histone proteins [27]. We also identified that the YEATS domain of the ENL protein is an important reader protein to interact with methacrylated proteins and mediate cellular functions [28]. The connections among valine metabolism, methacrylyl-CoA, and protein modifications warrant further investigation.

The goal of this study is to pursue the first investigation of cysteine *S*-2-carboxypropylation (C2cp) as a new post-translational modification (PTM) mark in the human proteome. Being an unexplored protein PTM, no antibody is available for C2cp detection in proteins using standard Western blot or immunoprecipitation methods. To circumvent this technical barrier, we designed *N*-propargyl methacrylamide (PMAA) as a bioorthogonal chemical probe to set up an antibody-free chemoproteomic platform for the detection and profiling of C2cp marks in the human proteome. Through quantitative and site-specific proteomic analysis, we have successfully identified more than 400 C2cp-containing protein targets and 100 modified cysteine sites. Functional annotation revealed that cysteine *S*-2-carboxypropylated proteins are involved in diverse essential cellular processes ranged from gene transcription, protein translation, RNA splicing, protein folding, to energy production. This study establishes C2cp as a new protein PTM and discloses an important molecular mechanism for understanding valine defect-induced metabolic diseases.

## 2. Results and Discussion

**Figure 1:**
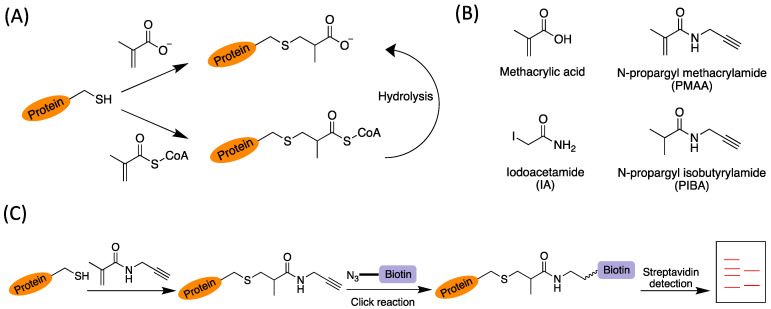
Design of chemical probes for *S*-2-carboxypropylated protein profiling. (**A**) Proposed pathway for the formation of cellular protein *S*-2-carboxypropylation. The protein can either directly conjugate with methacrylate or react with methacrylyl-CoA first and then be hydrolyzed to form *S*-2-carboxypropylation. (**B**) The structures of methacrylic acid, PMAA, IA, and PIBA. (**C**) Scheme for the probe PMAA to capture cellular *S*-2-carboxypropylated proteins. The cellular proteins were reacted with PMAA and further conjugated with biotin through a click reaction, after which the labeled proteins could be visualized through streptavidin detection (red lines signifying protein bands).

On account of the chemical reactivity of the methacrylyl group, we hypothesized that either methacrylyl-CoA or sodium methacrylate (Met-Na) could non-enzymatically react with the side-chain sulfhydryl group of cysteine residues in proteins to result in a thus far unstudied protein modification, cysteine *S*-2-carboxypropylation (C2cp) (Figure 1A). To investigate the possible modification of cysteine residues by methacrylate group on cellular proteins, we examined the reactions of methacrylyl-CoA with several thiol-containing biological compounds (cysteine, cysteamine, and coenzyme A). Conjugation products were easily detected with MALDI-MS which demonstrated that methacrylyl-CoA reacted efficiently with all of these biomolecules (Figure S1). These results were also consistent with a previous report through detection of free sulfhydryl groups by incubating the equimolar concentrations of methacrylyl-CoA with varied sulfhydryl compounds [20]. Furthermore, we tested the reactivity between methacrylyl-CoA and a cysteine-containing tripeptide, glutathione, and found it was able to be modified by methacrylyl-CoA (Figure S2). These results suggested that the direct chemical modification of proteins by methacrylyl-CoA is highly likely. In contrast, for the reactions between Met-Na and glutathione, no signals of adduct formation were detected through mass spectrometry (Figure S2). Therefore, the reactivity of methacrylyl-CoA was much higher than Met-Na. Furthermore, we sought to synthesize a methacrylic acid analog, N-propargyl methacrylamide (PMAA), as a bioorthogonal and competitive chemical probe to identify and profile *S*-2-carboxypropylated proteins (Figure 1B). We introduced a short amide bond in the probe for better biocompatibility and less steric hindrance. For comparison, we also synthesized a control compound, N-propargyl isobutyrylamide (PIBA), and used it to compete for protein labeling by the probe PMAA (Figure 1B). PMAA and PIBA were synthesized following the previously reported methods [29,30].

**Figure 2:**
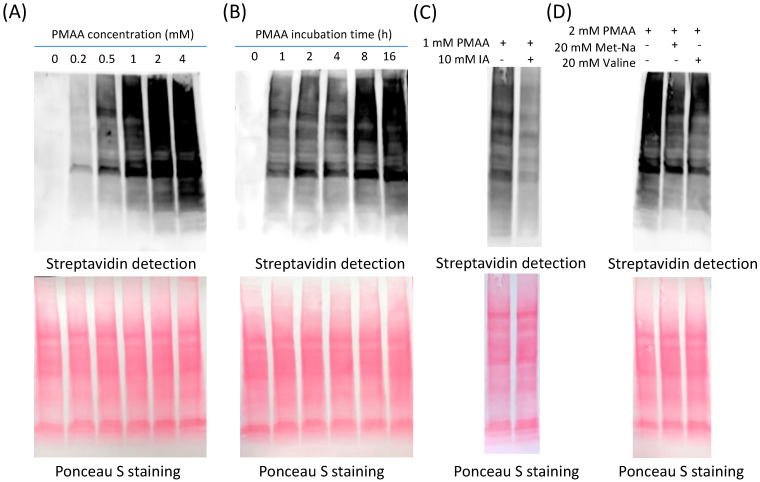
Evaluation of the probe PMAA labeling to detect *S*-2-carboxypropylation in native proteomes. (**A**) Concentration-dependent labeling of PMAA with cellular proteomes. HEK293T cellular proteins were incubated with probe PMAA with indicated concentration at 37 °C for 12 h, followed by CuAAC reaction to conjugate with biotin and detected by streptavidin-HRP. (**B**) Time-dependent labeling of PMAA with cellular proteomes. HEK293T cellular proteins were incubated with 2 mM PMAA at 37 °C for indicated time, followed by CuAAC reaction. (**C**) Using iodoacetamide (IA) as a competitive probe to block free cysteine. The HEK293T cellular proteins were pretreated with 10 mM IA for 2 h and then incubated with 1 mM PMAA for another 4 h (37 °C), followed by CuAAC reaction. (**D**) Competition of PMAA labeling by endogenous *S*-2-carboxypropylation induced by sodium methacrylate (Met-Na) and valine. HEK293T cells were incubated with 20 mM Met-Na or 20 mM valine separately, and the resulting cellular proteins were reacted with 2 mM PMAA at 37 °C for 12 h, followed by CuAAC reaction.

Next, we investigated how cellular proteins can be labeled by PMAA. Our idea is that PMAA reacts with cysteine residues in complex protein mixtures, after which the modified proteins can be biotin tagged via an alkyne click handle by conjugating with a biotin-azide reporter and then be detected by chemiluminescence (Figure 1C). In the experiment, cultured HEK293T cells were lysed and the whole lysate proteins were incubated with PMAA in a dose-dependent and a time-dependent manner. The labeled proteins were then reacted with biotin-azide (Click Chemistry Tools, Scottsdale, AZ, USA, catalog# 1265) through the copper-catalyzed azide–alkyne cycloaddition (CuAAC) reaction, resolved on SDS-PAGE, and imaged by streptavidin-HRP following the previous protocol [31,32]. As shown in Figure 2A, the whole cell lysates were effectively labeled with PMAA. The labeling became stronger with increasing concentrations of PMAA, and the labeling signals were detectable at as low concentration as 0.2 mM of this probe. Also, the labeling levels increased with prolonged incubation times in the range of 1–16 h (Figure 2B). Combined, incubation with 2 mM PMAA for 12 h would reach a saturating level of labeling on whole lysate proteins. We then proceeded to test whether the labeling resulted from the conjugation between the cysteine residues in cellular proteins and the alkene bond of the PMAA probe. As shown in Figure 2C, the PMAA labeling signal was abolished by the presence of iodoacetamide (IA), a commonly used alkylating agent that reacts with the thiol group of cysteines. In contrast, when either PIBA or sodium isobutyrate, which did not have a thiol-reactive functional group, was co-incubated in the mixture, there was no influence on the PMAA labeling (Figure S3A,B). These comparative results demonstrated that proteins were labeled by PMAA specifically through its thia-Michael addition reaction with the side-chain sulfhydryl group of cysteine residues (Figure 1C). We also tested whether PMAA could be used as a robust bioorthogonal probe to label proteins in different cellular systems. After incubating the probe with different types of cellular proteomes, strong protein labeling bands were observed in the whole lysate proteins from several cell lines tested, including a mouse embryonic fibroblast cell line (36T), colon cancer cell line (HCT116), and human epithelial cell line (HeLa) (Figure S4). Varied patterns of labeled proteins were seen from different cell lines, which suggests that protein C2cp modification profiles vary in different cells. To probe endogenous levels of protein *S*-2-carboxypropylation, we cultured HEK293T cells in the presence of 20 mM Met-Na or valine to boost cellular methacrylyl-CoA levels, and then the probe PMAA was used to label the cellular proteins and compete for the endogenous C2cp in proteins. As shown in Figure 2D and Figure S5, incubation of the cells with either 20 mM Met-Na or 20 mM valine decreased PMAA-driven labeling signals on the cellular proteins as compared with the cells with no treatment of Met-Na or valine. These results can be best explained by the fact that Met-Na and valine promoted endogenous cellular levels of methacrylate or methacrylyl-CoA, which led to enhanced protein C2cp modifications. Such enhanced protein C2cp modifications in cells reciprocally reduced the numbers of those cysteine sites that are accessible to conjugation with PMAA probe. These data demonstrated that PMAA can be applied as a competitive bioorthogonal chemical probe to profile C2cp substrates on cellular proteins.

**Figure 3:**
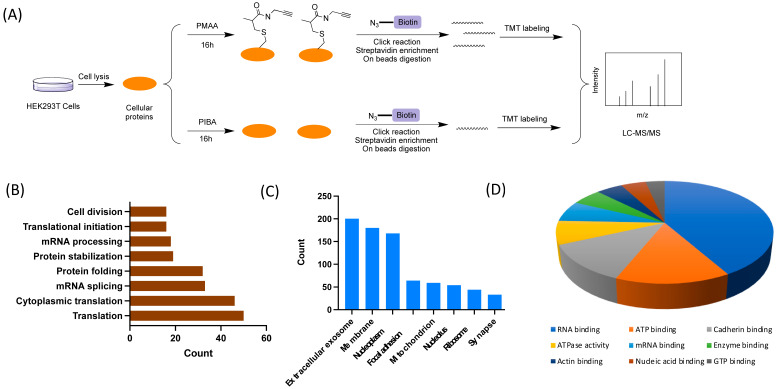
Quantitative proteomic profiling of *S*-2-carboxypropylated proteins by PMAA. (**A**) Schematic description of TMT-based multiplexed proteomic profiling of *S*-2-carboxypropylation in HEK293T cellular proteins by PMAA. (**B**) Biological process analysis of *S*-2-carboxypropylated proteins by gene ontology. (**C**) Cellular component analysis of *S*-2-carboxypropylated proteins in HEK293T cells. (**D**) Representative molecular function analysis of *S*-2-carboxypropylated proteins.

We next applied PMAA as a chemoproteomic probe to identify *S*-2-carboxypropylated proteins in HEK293T cells. The workflow is depicted in Figure 3A: HEK293T whole lysate proteins were prepared and then incubated with either 4 mM PMAA or 4 mM PIBA for 16 h at 37 °C, respectively. The protein samples were then precipitated with excess acetone and washed with ice-cold methanol to remove unreacted chemical probes. Thereafter, the labeled proteins were subjected to a CuAAC click reaction for conjugation with azide-diazo-biotin (Click Chemistry Tools, Scottsdale, AZ, USA, catalog# 1041) and then enriched on streptavidin beads. The affinity enriched proteins were cleaved from the beads with sodium dithionite (Na_2_S_2_O_4_) and then resolved on SDS-PAGE gel and imaged by silver staining. As expected, significantly more protein bands were shown in the group with PMAA incubation compared with the group with PIBA incubation (Figure S6). Next, we sought to globally identify the PMAA-labeled proteins using MS-based proteomics. To this end, the affinity-enriched proteins were subjected to on-bead trypsin digestion, and the resulting peptides were further labeled by tandem mass tags (TMTs) for multiplexed quantitative proteomic analysis (Figure 3A). The peptides labeled by the TMT reagents from different channels generated a unique reporter ion in the tandem MS, and the intensities of the reporter ions were used for quantifying peptides. To ensure the reliability of the results, we performed the replicate experiments and obtained a total of 1113 proteins (Table S1). After further setting the enrichment ratio cutoff at 1.4 and then selecting the proteins identified by more than two unique peptides, we finally narrowed the selection down to 403 proteins to be highly confident *S*-2-carboxypropylated protein substrates (Table S2).

To understand biological involvements and physiological functions of the identified C2cp proteins, we used the Database for Annotation, Visualization and Integrated Discovery (DAVID) to perform a Gene Ontology (GO) analysis of the 403 identified proteins [32]. The results showed that the PMAA-labeled proteins are involved in a plethora of biological processes including protein translation, mRNA splicing, protein folding, protein stabilization, mRNA processing, translational initiation, and cell division (Figure 3B). Cellular component analysis showed that a majority of the proteins are localized in the extracellular exosome, membrane, nucleoplasm, focal adhesion, and mitochondrion (Figure 3C), demonstrating a broad distribution of C2cp-modified proteins. Specifically, 59 proteins were found in the mitochondrion, including ALDH5A1, AIFM1, CS, HSPA9, and ACAT1, suggesting that C2cp may have multiple regulatory impacts on cellular metabolism through modifying mitochondrial proteins (Table S3). In addition, GO analysis based on molecular function indicated that a large number of the identified proteins mediate molecular bindings of RNA, ATP, cadherin, kinases, ATPases, actin, etc. (Figure 3D). Collectively, these functional annotation results suggest that protein C2cp modifications have a huge impact on diverse cellular pathways across all the major cellular organelles, which can be a rich source for further exploration of molecular mechanisms of methacrylyl-CoA and C2cp-regulated biological processes and pathological disorders.

**Figure 4:**
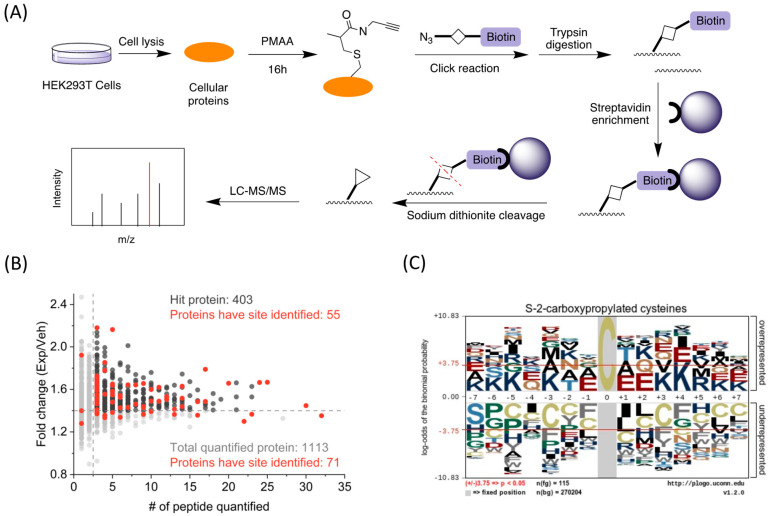
Identification of *S*-2-carboxypropylated cysteines by PMAA. (**A**) The working scheme for LC-MS/MS identification of *S*-2-carboxypropylation sites in HEK293T cells. (**B**) Identified *S*-2-carboxypropylated proteins and cysteine residues in HEK293T cells. The proteins which have Exp/Veh ratios > 1.4 and must be identified by more than two unique peptides are considered as hit proteins. (**C**) Sequence motif analysis of protein *S*-2-carboxypropylation. Images were generated with pLogo. The red horizontal lines on the pLogo plots denote *p* = 0.05 thresholds.

Next, we utilized the PMAA probe to globally identify C2cp sites on cellular proteins. The schematic is illustrated in the flow chart of Figure 4A: the cultured HEK293T cells were lysed and the whole lysate proteins were labeled by PMAA, conjugated with azide-diazo-biotin through CuAAC click reaction, and then subjected to trypsin digestion. The digested peptides were enriched using streptavidin agarose beads and eluted by sodium dithionite, which were then analyzed by LC-MS/MS for site identification. From two biological replicates, we obtained a total of 120 cysteine residues containing C2cp marks (Tables S4 and S5). Through further cross-checking with the 403 highly confident proteins mentioned above, we found 55 proteins had C2cp sites identified (Figure 4B). We also found eight proteins containing more than one site, including G3P, RS2, FLNA, STIP1, RS20, HNRNPU, TBB3, and FUBP2 (Table S6). To explore the structural features for C2cp, we analyzed the flanking amino acid sequences of the identified *S*-2-carboxypropylated cysteine residues by pLogo algorithm [33]. Specifically, we found positively charged lysine was significantly overrepresented at the −7, −6, −5, −3, +3, +4, +6, and +7 positions of *S*-2-carboxypropylation sites, whereas negatively charged glutamate was significantly overrepresented at the −1, +1, and +2 positions (*p* < 0.05, Figure 4C). In addition, glutamine, threonine, and arginine were highly enriched at the −4, −2, and +5 positions, respectively. The deprotonated Glu might possibly reduce the adjacent cysteine pKa through hydrogen bond formation, whereas a nearby arginine could elevate the cysteine pKa [34]. In contrast, we found Cys was generally underrepresented in the flanking sequences of the *S*-2-carboxypropylated sites. Therefore, the cysteine reactivity was greatly influenced by the local surrounding amino acids. It is noted that cysteine modification sites were also found in 40 proteins which were not identified through the global identification of PMAA-labeled proteins by MS-based proteomics, which highlights additional targets of cysteine *S*-2-carboxypropylation.

**Figure 5:**
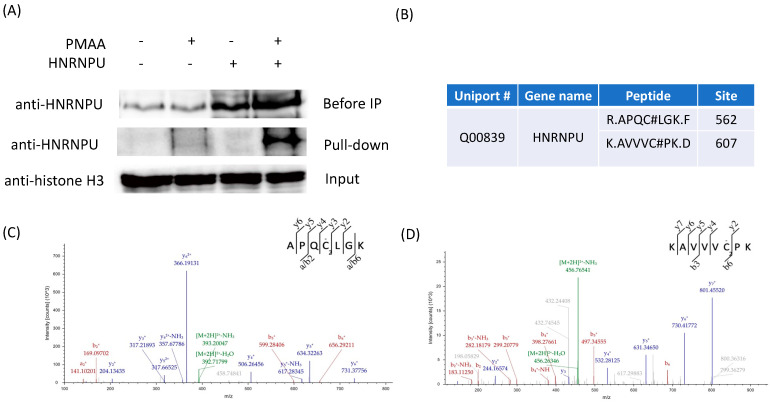
Validation of cysteine *S*-2-carboxypropylation on the selected protein, HNRNPU. (**A**) Verification of the PMAA-labeled protein (HNRNPU) by Western blot. HEK293T cell lysate proteins with or without HNRNPU overexpression were incubated with 2 mM PMAA for 12 h at 37 °C, and then the proteins were conjugated with azide-diazo-biotin through click reaction. After pull-down by streptavidin-beads, the eluted proteins were further detected by Western blot. (**B**) Identified C2cp sites on the protein HNRNPU by PMAA probe. (**C**) MS/MS peptide spectrum shows the C2cp modification on the C562 residue on HNRNPU. (**D**) MS/MS peptide spectrum shows the C2cp modification on the C607 residue on HNRNPU.

To corroborate the cysteine *S*-2-carboxypropylated proteins identified by our proteomic analysis, we selected the heterogeneous nuclear ribonucleoprotein U (HNRNPU) protein from our proteomic data list for biochemical validation. It has been found that the dysfunction of HNRNPU is related to neurodevelopmental syndromes, and that excess methacrylyl-CoA can also cause neurological disorders [18,35]. In the experiment, the HNRNPU plasmid (Addgene, Watertown, MA, USA, plasmid #35974) was transiently transfected into HEK293T cells and then the lysate protein mixture was labeled by PMAA, conjugated with azide-diazo-biotin, and enriched by streptavidin beads [36]. Thereafter, the labeled proteins were eluted by sodium dithionite. Enhanced-HNRNPU-labeling intensity was observed via Western blot when both the HNRNPU overexpression and PMAA probe were applied, which demonstrated that the intracellularly expressed protein HNRNPU was indeed labeled by PMAA, which was then pulled down by streptavidin beads (Figure 5A). Furthermore, we sought to confirm the identified C2cp sites on HNRNPU and investigated whether the modification was induced by Met-Na or methacrylyl-CoA. Based on our site-specific proteomic data, we identified two modification sites on HNRNPU by the probe PMAA, which were Cys562 and Cys607 (Figure 5B). We incubated the HNRNPU-overexpressed cell lysate proteins with either 1mM Met-Na or 1mM methacrylyl-CoA and then enriched the HNRNPU with anti-HNRNPU antibody. The obtained proteins were digested and further analyzed through LC-MS/MS. Using this approach, we successfully confirmed the two modification sites, Cys562 and Cys607, on HNRNPU with the treatment of methacrylyl-CoA (Figure 5C,D). On the other hand, with Met-Na incubation, we could not identify any C2cp sites, which coincided with the above observation that methacrylate is less reactive than methacrylyl-CoA. Together, these results demonstrated that PMAA was an excellent probe for profiling cellular protein C2cp, and the metabolite methacrylyl-CoA, but not Met-Na, was able to induce protein C2cp modifications.

## 3. Conclusions and Perspective

In this work, we have developed a highly efficient bioorthogonal chemical probe, PMAA, for the identification and profiling of *S*-2-carboxypropylated proteins in mammalian cells through chemical labeling, affinity enrichment, and proteomic identification. Using the probe, we showed that mammalian cellular proteins were extensively *S*-2-carboxypropylated and the modification was present in different cell lines such as 36T, HCT116, and HeLa cells. Using multiplexed quantitative proteomic analysis and site-specific profiling, we identified a total of 403 *S*-2-carboxypropylated proteins and 120 cysteine modification sites from HEK293T cells. To the best of our knowledge, this is the first direct demonstration and profiling of protein cysteine *S*-2-carboxypropylation (C2cp) on mammalian proteins. Furthermore, we experimentally validated cysteine *S*-2-carboxypropylation on the protein HNRNPU and confirmed the modification sites identified from the chemoproteomic profiling with the PMAA probe. Given the dysregulation of HNRNPU in neurodevelopmental syndromes, C2cp could be a potential regulatory biomarker in neurological disorders.

From the chemistry perspective, C2cp modification is a chemical conjugation of the thia-Michael addition between the nucleophilic sulfhydryl group on the side chain of cysteine residue and the electrophilic vinyl group of methacrylate, which we project is most likely non-enzymatic. We currently do not have experimental evidence to prove whether C2cp modification is reversible or dynamically regulated. From a chemical point of view, the C2cp modification is established via a thioether bond which is typically quite stable. Nevertheless, we remain open-minded as certain “eraser” enzymes might exist for the removal of this modification mark from proteins. Further efforts are warranted to characterize the possible mechanisms of protein C2cp modification in regulating cellular function. As cysteine residues are often sensitive to oxidative status, the addition of a C2cp mark to a target protein might be impactful to redox signaling, functioning as a molecular sensor for oxidative stress. By modifying the cysteine residue nearby or within an enzyme’s active site (a catalytic cysteine), the C2cp modification may act as an allosteric regulator or directly block the enzyme’s activity to mediate cellular functions.

Previously, using itaconate-alkyne, Qin et al. identified 1926 itaconated protein targets [37], whereas a lesser number of identified protein targets for *S*-2-carboxypropylated proteins were obtained in our experiment. This result, as well as different protein lists, suggests a different specificity for these two types of modifications. It could be that C2cp modification is more specific on cellular proteins compared with cysteine itaconation, or the chemical probes may have divergent reactivities in chemoproteomic profiling, particularly when cell types are different. Additionally, the lower number of identified C2cp proteins in our experiment might suggest a short lifetime for this modification.

Interestingly, we found that the C2cp mark was far more effectively induced by the metabolite methacrylyl-CoA rather than Met-Na, which demonstrated that methacrylyl-CoA is a more reactive metabolite than Met-Na in protein modification. Since methacrylyl-CoA is a key metabolic intermediate in the valine degradation pathway, C2cp marks in proteins may represent a new regulatory mechanism for valine-associated metabolic diseases. Further studies are needed to investigate the detailed mechanisms around the influence of the C2cp modification on valine-pertinent disease processes.

Interestingly, other types of cysteine carboxyalkylations were recently observed by Wang et al. [38], which conceptually aligns well with our findings reported herein. Their studies reported several species of cysteine carboxyalkylation on proteins which were driven by reactive metabolites from fatty acid metabolism. Our study revealed that C2cp is a particular cysteine carboxyalkylation mark derived from valine catabolism. It is possible that cysteine carboxyalkylation is a type of versatile PTM in the proteome that has been overlooked in the literature.

Overall, our work identified cysteine *S*-2-carboxypropylation in mammalian proteins and mapped out its proteomic distribution in the proteome. Additionally, the PMAA chemical probe we developed in this present study would serve as a useful tool for studying other cysteine-participating protein modifications.

## Data Availability

The original contributions presented in this study are included in the article and Supplementary Material. Further inquiries can be directed to the corresponding author.

## Supplementary Materials

The following supporting information can be downloaded at: https://www.mdpi.com/article/10.3390/molecules30214255/s1: experimental methods and additional figures. Figure S1: Mass spectra of MC-CoA reacting with cysteine, cysteamine, or coenzyme A; Figure S2: Mass spectra of MC-CoA or sodium methacrylate reacting with glutathione; Figure S3: Competitive labeling of PMAA by PIBA and sodium isobutyrate; Figure S4: Evaluation of the probe PMAA labeling in different cell lines; Figure S5: Competitive labeling of PMAA by the endogenous S-2-carboxypropylation; Figure S6: Enrichment of the PMAA labeled protein.
