# Supplementary material for: Discovery and Profiling of Protein Cysteine S-2-Carboxypropylation
Source: Molecules. 2025 Oct 31;30(21):4255. doi: 10.3390/molecules30214255 (PMC12608796; doi:10.3390/molecules30214255)
Supplement: Supplementary file 1 [file molecules-30-04255-s001.zip › molecules-3919662-Supplementary Materials.zip!/molecules-3919662-Supplementary Materials.pdf]

Supplementary Information

Discovery and Profiling of Protein Cysteine *S*-2-Carboxypropylation

Jiabao Song[†], Kejun Yin[≠], Ronghu Wu[≠], Y. George Zheng[†]*

[†]Department of Pharmaceutical and Biomedical Sciences, College of Pharmacy, University of Georgia, Athens, GA 30602.

[≠]School of Chemistry and Biochemistry, Georgia Institute of Technology, Atlanta, GA 30332.

*Corresponding author. Email: yzheng@uga.edu

Supplementary figures

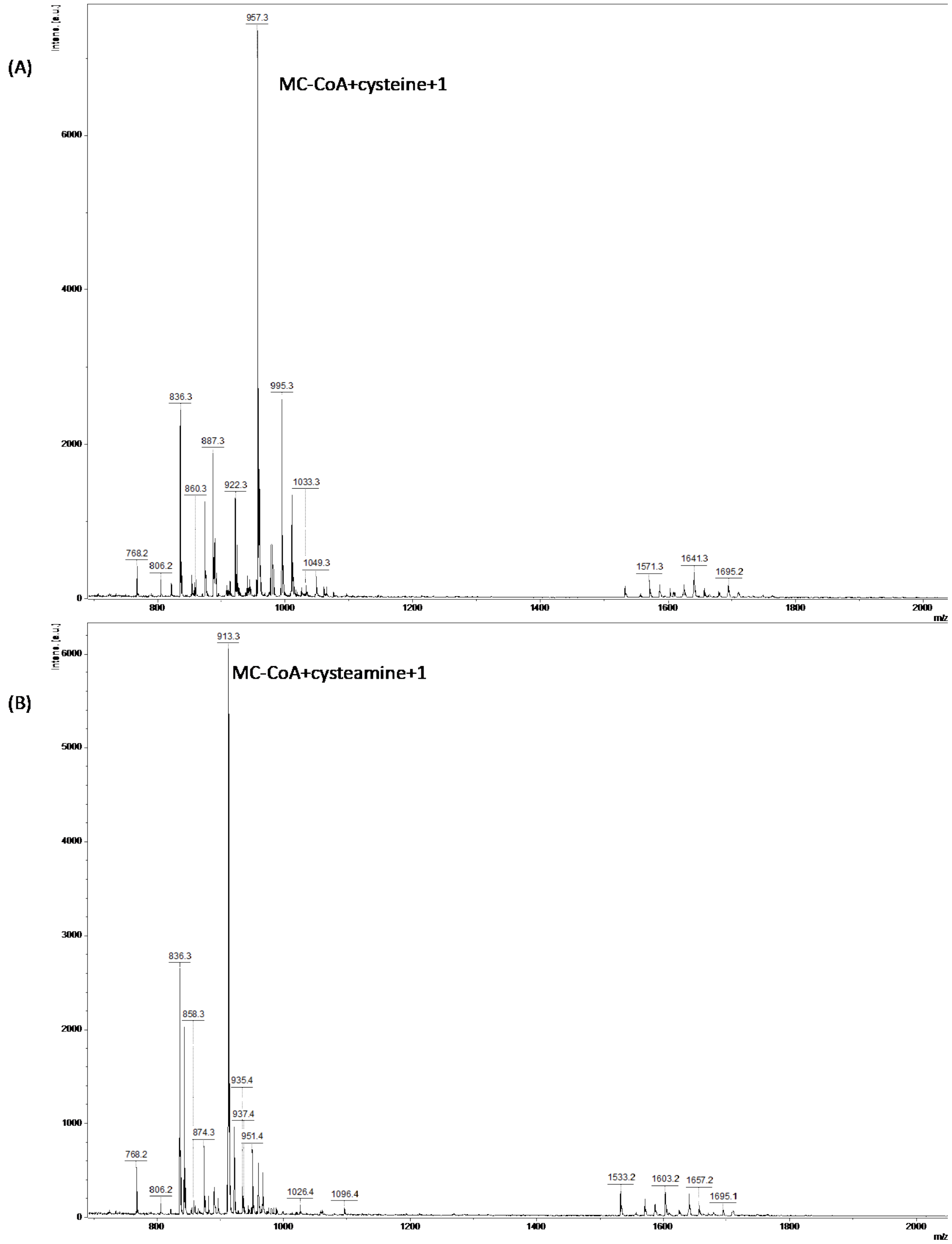

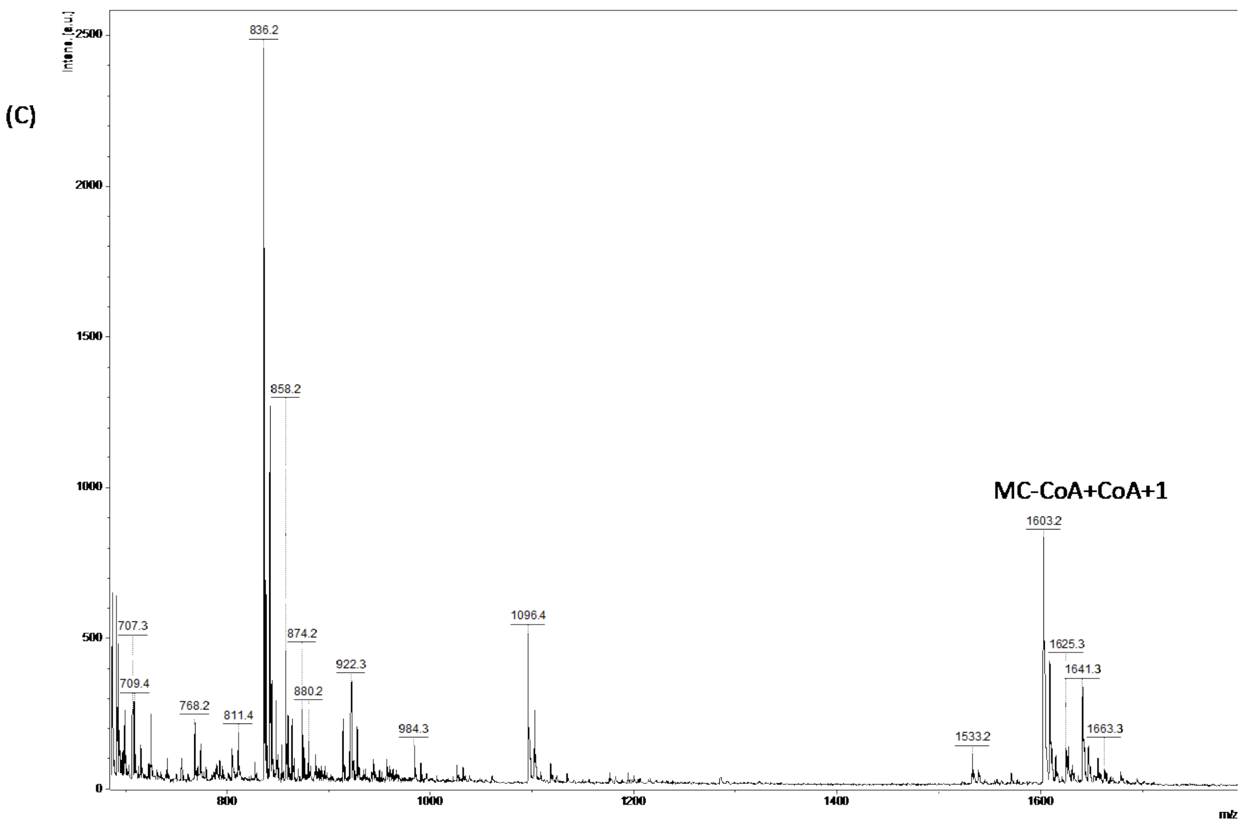

Supplementary Figure S1.

Mass spectra of MC-CoA reacting with cysteine, cysteamine, or coenzyme A. (A) 1 mM MC-CoA reacted with 1 mM cysteine at 37 °C for 4 h. (B) 1mM MC-CoA reacted with 1mM cysteamine at 37 °C for 4 h. (C) 1mM MC-CoA reacted with 1mM coenzyme A at 37 °C for 4 h.

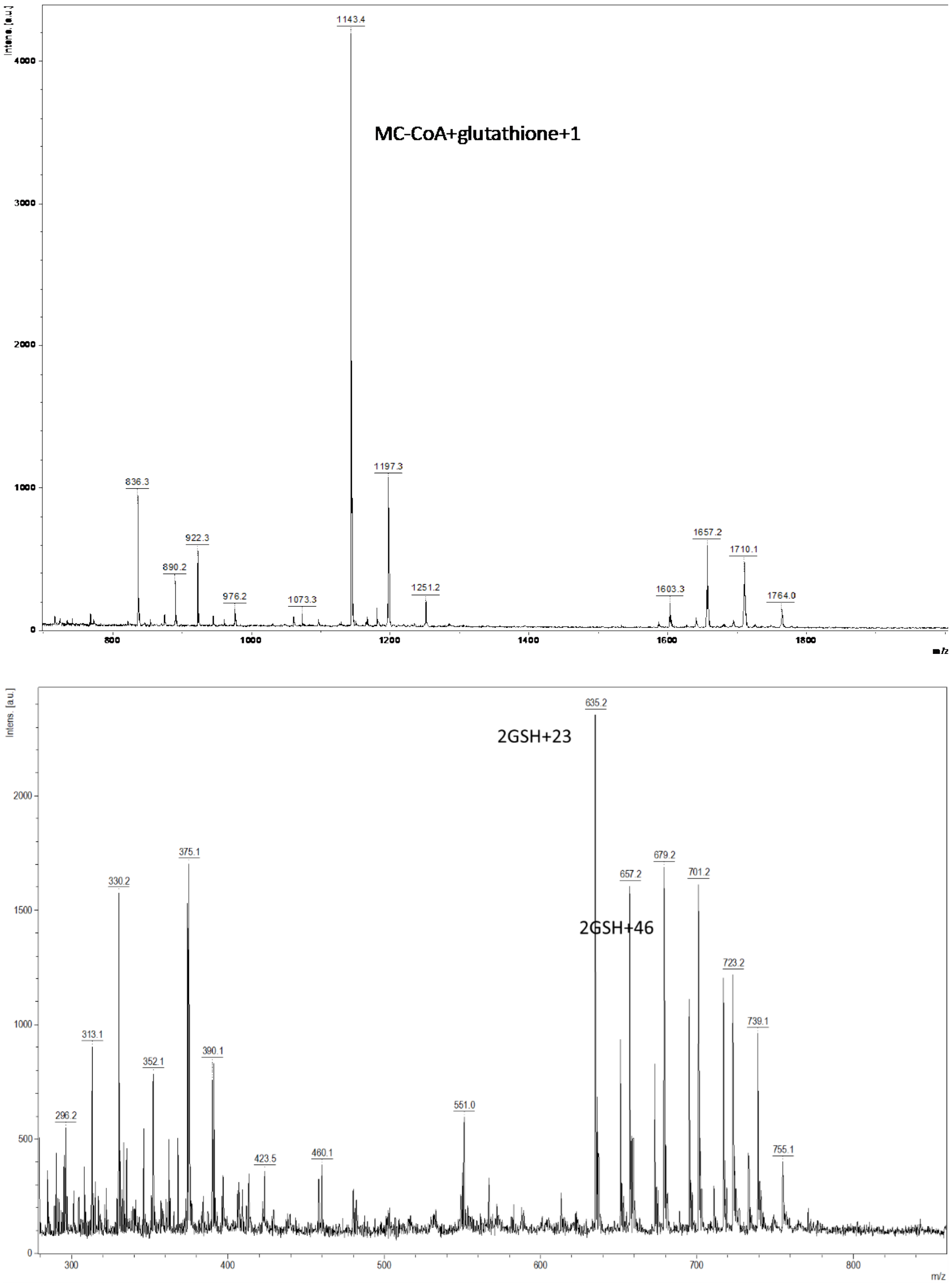

Supplementary Figure S2.

Mass spectra of MC-CoA or sodium methacrylate reacting with glutathione.
Upper: Mass spectra of the mixture of MC-CoA reacting with glutathione. 1mM MC-CoA reacted with 1mM glutathione at 37 °C for 4 h.

Lower: Mass spectra of the mixture of sodium methacrylate reacting with glutathione. No conjugation product was found. Only the mass peaks for GSH were seen.

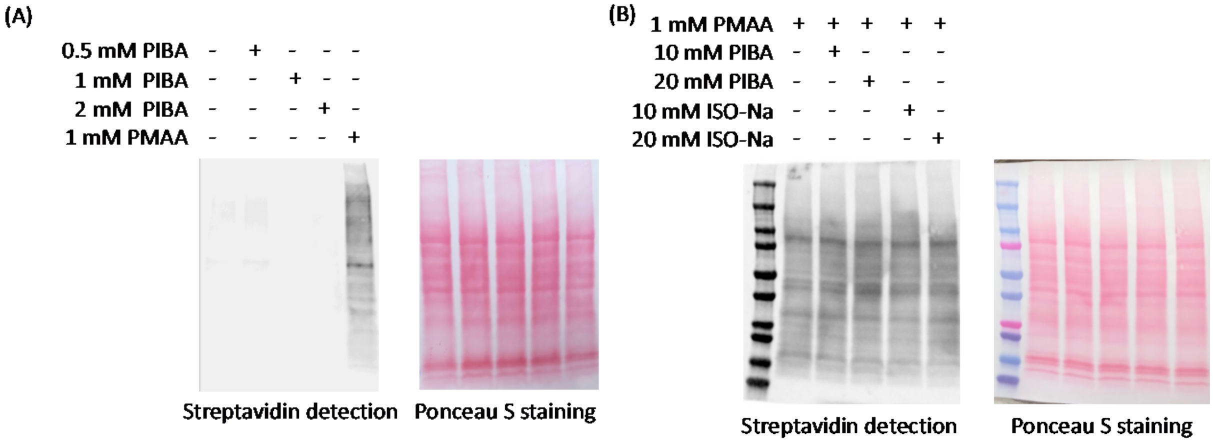

Supplementary Figure S3.

Competitive labeling of PMAA by PIBA and sodium isobutyrate. (A) The HEK293T cell lysate protein was incubated with indicated concentration of the probe PIBA or PMAA at 37 ℃ for 4 h. (B) The HEK293T cell lysate protein was pretreated with PIBA or sodium isobutyrate for 2 h and then incubated with 1 mM PMAA at 37 ℃ for another 4 h.

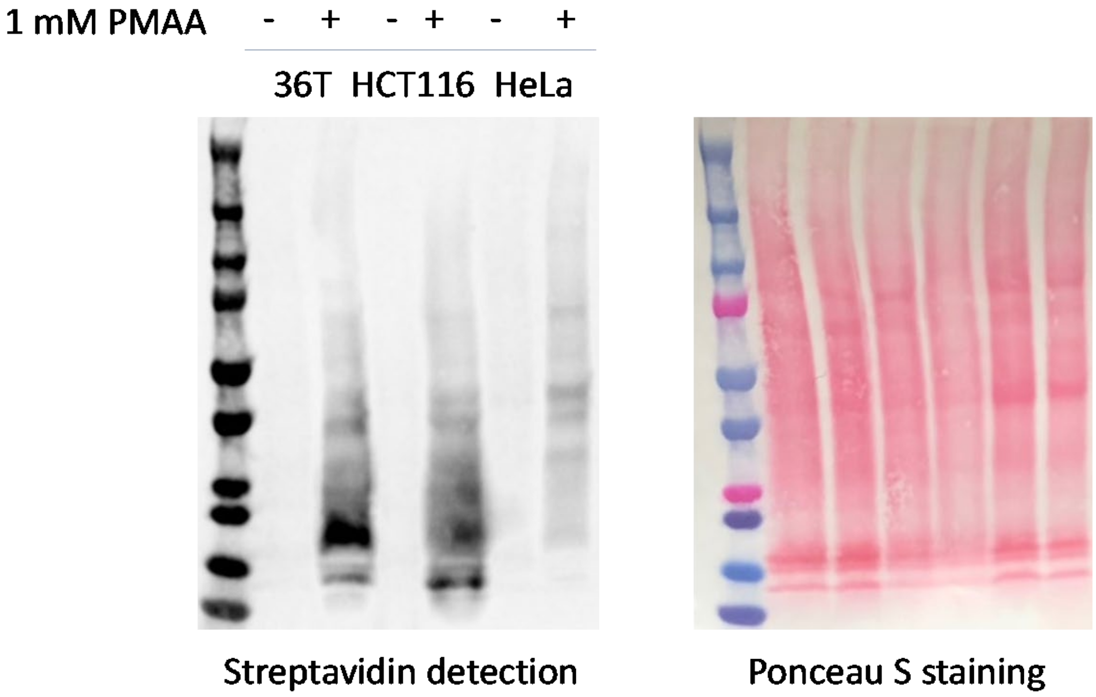

Supplementary Figure S4.

Evaluation of the probe PMAA labeling in different cell lines. The cellular protein extracted from 36T, HCT116 and Hela cells were reacted with 1 mM PMAA at 37 °C for 12 h.

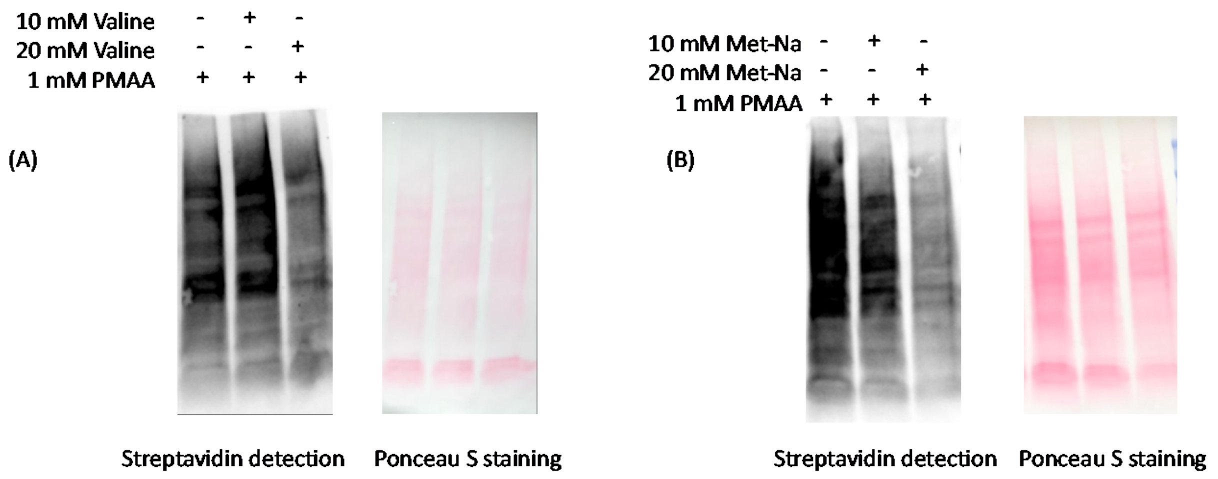

Supplementary Figure S5.

Competitive labeling of PMAA by the endogenous *S*-2-carboxypropylation. (A) HEK293T cells were incubated with 10 mM or 20 mM valine at 37 °C for 24 h, and then the cellular proteins were reacted with 1 mM PMAA at 37 °C for 12 h. (B) HEK293T cells were incubated with 10 mM or 20 mM sodium methacrylate at 37 °C for 24 h, and then the cellular protein were reacted with 1 mM PMAA at 37 °C for 12 h.

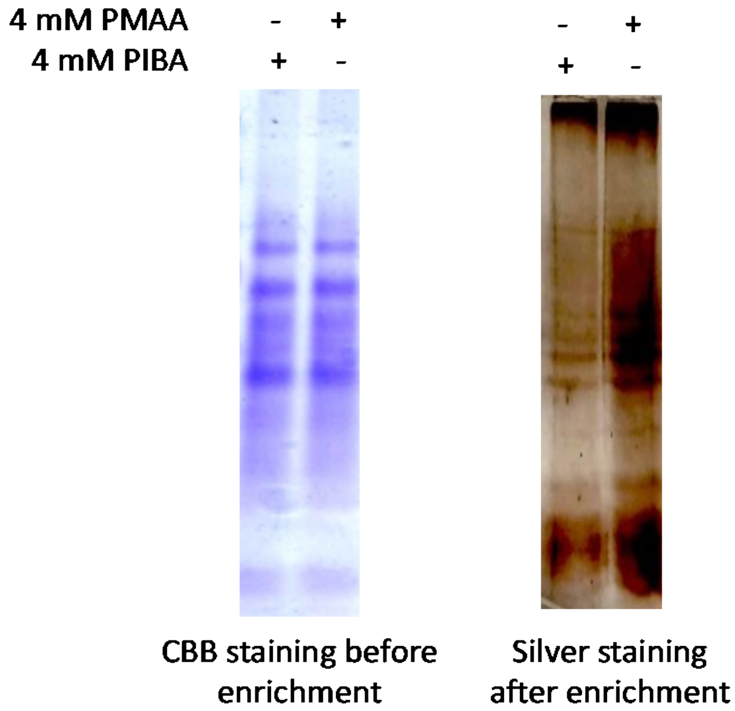

Supplementary Figure S6.

Enrichment of the PMAA labeled protein. The HEK293T cellular protein were reacted with 4 mM PMAA or 4 mM PIBA at 37 °C for 16 h , and then the labeled protein were further reacted with azide-diazo-biotin and pull-down by streptavidin beads. The eluted protein were then imaged by silver staining.

Experimental methods

Reagents and materials

Unless otherwise noted, all chemicals and solvents were purchased from Sigma–Aldrich and used without further purification. Click chemistry reagents biotin-azide (catalog# 1265) and azide-diazo-biotin (catalog# 1041) were purchased from Click Chemistry Tools. Protein dual color standards and precast protein gels (4%–20%) were purchased from Bio-Rad Laboratories. High sensitivity streptavidin-HRP was purchased from Thermo Fisher Scientific (catalog# 21130). High-capacity streptavidin agarose was purchased from Thermo Fisher Scientific (catalog# 20359). Plasmid hnRNPU (pcDNA3.1-hnRNPU-V5) was a gift from Susana Valente (Addgene plasmid # 35974; http://n2t.net/addgene:35974; RRID: Addgene_35974).[1] The anti-hnRNPU antibody (catalog# sc-32315) was purchased from Santa Cruz. The anti-Histone H3 antibody (catalog# sc-517576) was purchased from Santa Cruz. The anti-mouse-HRP linked antibody (catalog# 7076) was purchased from Cell Signaling. HEK293T, Hela and HCT 116 cells were purchased from

American Type Culture Collection (ATCC). MEF cell line (36T cells) was a gift from Dr. Mark Parthun's lab.

Synthesis of PMAA, PIBA and MC-CoA

The probe N-propargyl methacrylamide (PMAA) was synthesized as previously described.[2] The probe N-propargyl isobutyrylamide (PIBA) was synthesized following the previous protocol.[3] methacrylyl-CoA (MC-CoA) was synthesized as described in our previous publication.[4]

MALDI-mass analysis of the reactions between MC-CoA and thiol-containing biological compounds

1mM MC-CoA was used to react with 1 mM cysteine, cysteamine, or coenzyme A at 37 ℃ for 4 h in PBS, respectively. The resulted samples were sent for MALDI mass detection.

The same condition was used for the reaction between MC-CoA and cysteine-containing peptide, glutathione.

Cell culture and preparation of whole cellular protein

HEK293T, Hela, 36T, and HCT116 cells were cultured in Dulbecco's Modified Eagle Medium (DMEM). The medium was supplemented with 10% fetal bovine serum (FBS, Life Technologies) and 1% penicillin-streptomycin (Thermo Fisher Scientific, #15140122). All cells were maintained in a humidified incubator at 37°C with 5% CO2.

To extract whole cellular proteins, cells were first cultured to approximately 90% confluence. The cells were then washed three times with PBS, scraped, and harvested. Cell pellets were collected by centrifugation (5000 rpm, 5 min, 4°C). Pellets were resuspended in ice-cold M-PER mammalian protein extraction reagent (ThermoFisher Scientific, #78501) fortified with 1% protease inhibitor cocktail (ThermoFisher Scientific, #78438). Following a 15-minute incubation on ice, the lysate was sonicated (30% amplitude) to release total cellular proteins. Insoluble debris was pelleted by high-speed centrifugation (13200 rpm, 20 min, 4°C). The resulting supernatant, containing the soluble cellular proteins, was collected, and its protein concentration was determined via the Bradford assay.

Protein labeling by the probe PMAA, CuAAC reaction, and streptavidin detection

The methods for labeling, CuAAC reaction and streptavidin detection were adapted from the previous experimental procedures.[5] To label proteins, 50 µg of the cellular proteome was incubated with the PMAA probe (at indicated concentrations) for 12 hours at 37°C. Following incubation, proteins were precipitated by adding 5 volumes of ice-cold acetone. The precipitate was pelleted (5000 g, 10 min, 4°C) and washed three times with ice-cold methanol. The resulting protein pellet was resuspended in PBS containing 0.4% SDS. The resuspended proteins were then subjected to a CuAAC reaction by adding a click cocktail containing 50 µM biotin-azide, 2.5 mM sodium ascorbate, 0.5 mM copper sulfate, and 0.25 mM BTTAA ligand. After a 1-hour incubation at room temperature, the reaction was quenched with protein loading dye, and samples were boiled for 5 minutes. Samples were then loaded onto a 4-20% SDS-PAGE gradient gel. After electrophoresis, proteins were transferred to a nitrocellulose membrane. The membrane was blocked for 1 hour with 5% non-fat milk, followed by a 1-hour incubation with streptavidin-HRP. Finally, the blot was visualized using a chemiluminescence scanner.

Protein enrichment and protein digestion for protein identification

Lyophilized lysates were first reconstituted in 100 mM DPBS. Separately, streptavidin beads were washed twice with 100 mM DPBS to equilibrate them. The reconstituted lysates were then incubated with the washed beads for 2 hours at room temperature. To remove non-specific binding proteins, the beads were transferred to a Thermo spin column and washed ten times with 100 mM DPBS.

The beads, containing the enriched proteins, were resuspended in a digestion buffer (50 mM HEPES pH 8.1, 1.6 M urea). Trypsin (Promega) was added at a 50:1 (w/w) ratio, and the digestion proceeded for 16 hours at 37°C with shaking. Following digestion, the beads were removed using a spin column. The reaction in the collected supernatant was quenched by adding trifluoroacetic acid (TFA) to a final concentration of 0.4%. The acidified digests were then centrifuged, and the resulting peptides were desalted using 50 mg SepPak tC18 cartridges (Waters).

Protein digestion and peptide enrichment for sites identification

Lyophilized lysates were transferred to new tubes and resuspended in the digestion buffer containing 50 mM HEPES pH=8.1, 1.6 M urea, and proteins were digested with 50:1 (w/w) trypsin (Promega) for 16 h at 37 ˚C with shaking. The digestion was quenched by adding trifluoroacetic acid (TFA) to a final concentration of 0.4%. Digests were centrifuged and desalted using 50 mg SepPak tC18 cartridges (Waters) before lyophilization.

For each sample, 50 μL Neutravidin beads (Thermo) were used to enrich biotinylated peptides. Beads were washed 3 times by 100 mM DPBS, then incubated with peptides dissolved in 500 μL 100 mM DPBS for 2 h. Unbounded peptides were removed through 10 times washes with 100 mM DPBS, and tagged peptides were eluted in 25 mM sodium dithionite solution for 30 min twice. Two elutes were combined and desalted as mentioned above.

TMT labeling and peptide fractionation by HPLC

Purified peptides were first lyophilized, then dissolved in 100 μL of 100 mM HEPES buffer (pH 8.5) together with 30 μL acetonitrile (ACN). Labeling was carried out using the TMT6plex reagent (Thermo), and the reaction was allowed to proceed for one hour. To terminate excess labeling reagent, 10 μL of 5% hydroxylamine was added. The labeled peptide mixtures were combined, cleaned, and separated into three fractions with 20%, 50%, and 80% ACN. Each fraction was subsequently subjected to additional purification using the StageTip protocol.

LC-MS/MS analysis for proteomic identification

The lyophilized peptide samples were reconstituted in 6 μL of loading buffer containing 5% ACN and 4% formic acid (FA). From this, 3 μL was injected onto a microcapillary column (75 μm × 16 cm) packed with C18 resin (Magic C18AQ, 5 μm, 200 Å) using a Dionex WPS-3000TPLRS autosampler (UltiMate 3000 Thermostatted Pulled Loop Rapid Separation Wellplate Sampler). Peptide separation was achieved by reversed-phase HPLC on an UltiMate 3000 binary pump with a 150-minute gradient optimized for TMT-labeled peptides. Mass spectrometric analysis was performed on an LTQ Orbitrap Elite hybrid dual-cell quadrupole linear ion trap–Orbitrap instrument (Thermo Scientific) operated with Xcalibur 3.0.63 software. Data were acquired in a data-dependent Top15 mode: each cycle began with a full MS scan in the Orbitrap (resolution 60,000; AGC target 1 × 10^6), followed by MS/MS of the 15 most intense precursor ions. Dynamic

exclusion: 90s, charge state: 2-6. For MS2 spectra, higher-energy collisional dissociation (HCD): 30 normalized collision energy (NCE); isolation width: 1.2 m/z; resolution: 15,000.

Database searching, data filtering, and protein quantification

Raw spectra were converted to mzXML format and searched with the SEQUEST algorithm (version 28) (Eng et al., 1994) against the UniProt human proteome database (Homo sapiens, https://www.uniprot.org/taxonomy/9606). To estimate false discovery rates (FDR), each protein sequence was included in both forward and reversed orientations. Search parameters were as follows: precursor mass tolerance of 10 ppm; fragment ion mass tolerance of 0.025 Da; trypsin specificity with up to two missed cleavages; variable modification of cysteine (+301.153874); and fixed TMT modifications on lysine residues and peptide N-termini (+229.1630).

Peptide and protein identifications were validated using the target-decoy approach (Elias and Gygi, 2007). Linear discriminant analysis (LDA) was applied to separate correct from incorrect identifications based on features such as XCorr, ΔCn, and precursor mass error. Peptides shorter than seven amino acids were excluded, and the dataset was filtered to achieve.

Quantification was based on TMT reporter ion intensities extracted from MS2 spectra. Isotopic correction factors provided by Thermo were applied, and protein-level intensities were calculated by summing the signals of all unique peptides assigned to each protein.

To assess site localization confidence, a ModScore was computed for each cysteine modification site. This probabilistic scoring algorithm evaluates all cysteines within a peptide by comparing fragment ions unique to each possible site. Sites with ModScore > 13 ($p < 0.05$) were considered confidently localized. For quantification, only peptides containing at least one confidently localized cysteine site were included. Since cysteine residues are relatively uncommon, most peptides contained only a single identifiable site.

Bioinformatic analysis

Biological process, cellular component, and molecular function analysis of the identified *S*-2-carboxypropylated proteins were carried out through DAVID bioinformatics.[6] Sequence preference motif was generated by pLogo algorithm.[7]

Western blotting analysis

For Western blot analysis, enriched and input samples were resolved by SDS-PAGE and transferred to a nitrocellulose membrane. The membrane was blocked for 1 hour with 5% non-fat milk. Subsequently, the membrane was incubated overnight at 4°C with either anti-HNRNPU or anti-Histone H3 primary antibodies. After three 5-minute washes with TBST (10 mM Tris pH 8.0, 150 mM NaCl, 0.5% Tween 20), the membrane was incubated with the appropriate HRP-conjugated secondary antibody for 1 hour at room temperature. Following three final 5-minute washes in TBST, the proteins were visualized using an enhanced chemiluminescence (ECL) detection system according to the manufacturer's protocols and imaged with a chemiluminescence scanner.

Transfection, PMAA labeling and enrichment of the protein HNRNPU

HEK293T cells were plated and cultured until 70% confluence and the transient transfection of the plasmid hnRNPU (pcDNA3.1-hnRNPU-V5, plasmid# 35974, addgene) was conducted by Lipofectamine 3000 (ThermoFisher SCIENTIFIC, Product #L3000008) according to the

manufacture's protocol followed by 48 h of incubation. The whole cellular proteins were prepared following the aforementioned procedure.

PMAA labeling of the protein HNRNPU followed the above-described protocol. Briefly, 100 μg cellular proteins were incubated with 2 mM PMAA at 37 °C for 12 h. The whole proteins were then precipitated with 5-fold ice-cold acetone and the precipitated proteins were collected by centrifugation at 5000 g for 10 min at 4 °C, washed by ice-cold methanol for three times. The resulting proteins were resuspended by PBS with 0.4% SDS. The protein solutions were then subjected to CuAAC reaction. For CuAAC reaction, the proteins were mixed with click cocktail containing 50 μM azide-diazo-biotin, 2.5 mM sodium ascorbate, 0.5 mM copper sulfate and 0.25 mM ligand BTTAA. After 1 h incubation at room temperature, the proteins were further precipitated to remove excess azide-diazo-biotin and then redissolved in PBS with 0.2% SDS.

High-capacity streptavidin agarose was first equilibrated by PBS and then incubated with the protein sample for 1 h with gentle agitation. Next, the resins were collected by centrifugation at 5000g for 5 minutes and washed with PBS supplemented with 0.2% (w/v) SDS, PBS supplemented with 0.1% (w/v) of SDS and 6M urea and 50 mM of NH_4HCO_3 supplemented with 0.1% of SDS. Then, the resins were incubated with 25 mM of $Na_2S_2O_4$ in 50 mM NH_4HCO_3 for 1 h to elute the protein off the beads. The mixture was then centrifuged at 5000g for 5 minutes and the supernatant was collected and dried by SpeedVac and redissolved in 100 μL SDS loading buffer. The solution was then subjected to SDS-PAGE running and western blotting analysis.

Protein reaction and Immunoprecipitation of the protein HNRNPU

HNRNPU overexpression and the whole cellular protein extraction were following the aforementioned procedures. 100 μg HNRNPU overexpressed cellular proteins were incubated with either 1 mM sodium methacrylate or 1mM MC-CoA at 37 °C for 12 h. The resulted samples were subjected to immunoprecipitation for HNRNPU enrichment by Protein G Plus-agarose (Santa Cruz Biotechnology, Product# sc-2002) following the standard protocol. The enriched proteins were separated by SDS-PAGE and the gel bands were cut for LC-MS/MS analysis.

In gel digestion, LC-MS/MS analysis of the modification sites on the protein HNRNPU

Gel bands were excised and destained with two washes of 50% acetonitrile (ACN) in 20 mM ammonium bicarbonate (AmBic) (~pH 7.5-8). The gel pieces were then dehydrated with 100% ACN and dried in a SpeedVac concentrator. The dried pieces were rehydrated on ice with trypsin solution (10 ng/μL in 20 mM AmBic) until saturated. After overnight digestion at 37°C, tryptic peptides were extracted twice using 50% ACN / 0.1% formic acid (FA). The pooled extracts were dried in a SpeedVac.

Mass spectrometry was performed on an LTQ Orbitrap Elite (Thermo Fisher Scientific) coupled to a Proxeon Easy NanoLC system. Peptides were loaded onto a self-packed C18 reversed-phase column (200 Å, 5 μM Bruker MagicAQ resin) and eluted at 450 nL/min. The gradient used Buffer A (0.1% FA) and Buffer B (0.1% FA in 99.9% ACN) as follows: 0% B for 2 min, 0-30% B over 50 min, 30-50% B over 10 min, and 50-95% B over 10 min. Data were acquired in data-dependent acquisition (DDA) mode. A full MS1 survey scan (120,000 resolution) was followed by CID MS/MS analysis (15,000 resolution) of the top 10 most abundant precursor ions. The instrument was controlled by Xcalibur software (v2.2). Raw data were processed using Proteome Discoverer (v1.4) and the Mascot search engine (v2.7) against the Uniprot database. All spectra corresponding to potential modifications were manually inspected to verify assignment accuracy.

References

1. Mediouni, S.; Jablonski, J.; Paris, J. J.; Clementz, M. A.; Thenin-Houssier, S.; McLaughlin, J. P.; Valente, S. T., Didehydro-cortistatin A inhibits HIV-1 Tat mediated neuroinflammation and prevents potentiation of cocaine reward in Tat transgenic mice. *Curr HIV Res* **2015,** 13, (1), 64-79.

2. Yu, F.; Wang, Y. Z.; Hang, Y.; Tang, W. M.; Zhao, Z. F.; Oupicky, D., Synthesis and biological characterization of clicked chloroquine copolymers as macromolecular inhibitors of cancer cell migration. *Journal of Polymer Science Part a-Polymer Chemistry* **2019,** 57, (22), 2235-2242.

3. Krzywik, J.; Nasulewicz-Goldeman, A.; Mozga, W.; Wietrzyk, J.; Huczynski, A., Novel Double-Modified Colchicine Derivatives Bearing 1,2,3-Triazole: Design, Synthesis, and Biological Activity Evaluation. *Acs Omega* **2021,** 6, (40), 26583-26600.

4. Delaney, K.; Tan, M.; Zhu, Z.; Gao, J.; Dai, L.; Kim, S.; Ding, J.; He, M.; Halabelian, L.; Yang, L.; Nagarajan, P.; Parthun, M. R.; Lee, S.; Khochbin, S.; Zheng, Y. G.; Zhao, Y., Histone lysine methacrylation is a dynamic post-translational modification regulated by HAT1 and SIRT2. *Cell Discov* **2021,** 7, (1), 122.

5. Song, J.; Han, Z.; Zheng, Y. G., Identification and Profiling of Histone Acetyltransferase Substrates by Bioorthogonal Labeling. *Curr Protoc* **2022,** 2, (7), e497.

6. Huang da, W.; Sherman, B. T.; Lempicki, R. A., Systematic and integrative analysis of large gene lists using DAVID bioinformatics resources. *Nat Protoc* **2009,** 4, (1), 44-57.

7. O'Shea, J. P.; Chou, M. F.; Quader, S. A.; Ryan, J. K.; Church, G. M.; Schwartz, D., pLogo: a probabilistic approach to visualizing sequence motifs. *Nat Methods* **2013,** 10, (12), 1211-2.
